# Book Notices

**Published:** 1880-06-20

**Authors:** 


### *BOOK NOTICES.*

STUDENT'S POCKET MEDICAL LEXICON: Giving the correct pronunciation and definition of all words, and terms in general use in Medicine and the Collateral Sciences: with an appendix containing a list of poisons and their antidotes, abbreviations used in prescriptions, and a metric scale of doses. By Elias Longley, author of pronouncing Vocabulary of Geographical and Personal Names, Eclectic Manual of Phonography, etc. Philadelphia—Lindsay & Blakiston, 1879.

This is an exceedingly valuable and useful work for the student of Medicine.

THE MICROSCOPE AND MICROSCOPICAL TECHNOLOGY: A Text Book for Physicians and Students, by Heinrich Frey, Professor of Medicine in the University of Zunct. Translated and Edited by George R. Cutter, M.D., Surgeon in New York Eye and Ear Infirmaary, Ophthalmic and Aural Surgeon to the St. Catherine and Williamsborough Hospitals, etc. Illustrated by three hundred and eighty engravings on wood: Second edition. New York, William Wood & Co., 1880.

This is a large volume of 606 octavo pages, neatly gotten up in bold plain type. The style is excellent, though the translation from the German is very literal. The work is able and comprehensive, treating of theory and mechanism of the Microscope-Apparatus for measuring and drawing; testing and using the Microscope; preparation of Microscopic objects; method of staining, etc. The blood, lymph, chyle, mucus and pus, etc., etc., described and illustrated with the various tissues and organs of the body. We have not space to detail or do justice to this valuable work. It should be in the Library of every Student and progressive Medical man.

A PRACTICAL TREATISE ON NERVOUS EXHAUSTION (Nurasthemia): its symptoms, nature, sequences, treatment, by George M. Beard, A.M., M D. Fellow of the New York Academy of Medicine; Vice President of the American Academy of Medicine; Member of the American Neurological Association; of the American Association, the New York Neurological Society. New York, William Wood & Co., 1880.

This is a work of 103 octavo pages, containing much interesting matter upon a class of affections little understood by a majority of practitioners. Nervous affections of the character herein described are becoming more and more frequent in this country, and therefore the more important to be known by the practitioner. The work is very instructive, and will well repay perusal.

